# Educational Level Is Related to Physical Fitness in Patients with Type 2 Diabetes – A Cross-Sectional Study

**DOI:** 10.1371/journal.pone.0164176

**Published:** 2016-10-12

**Authors:** Lara Allet, Olivier Giet, Jérôme Barral, Nicolas Junod, Dominique Durrer, Francesca Amati, Gerasimos P. Sykiotis, Pedro Marques-Vidal, Jardena J. Puder

**Affiliations:** 1 Department of Physiotherapy, School of Health, University of Applied Sciences of Western Switzerland, Geneva, Switzerland; 2 Department of Community Medicine, University Hospitals and University of Geneva, Geneva, Switzerland; 3 Service de Physiothérapie, Hôpital de Fribourg, Fribourg, Switzerland; 4 Research Group of Institute of Sport Sciences University of Lausanne (GRISSUL), University of Lausanne, Lausanne, Switzerland; 5 Service of Endocrinology, Diabetes and Metabolism, Centre Hospitalier Universitaire Vaudois, University of Lausanne, Lausanne, Switzerland; 6 Eurobesitas Centre, Vevey, Switzerland; 7 Department of Physiology, University of Lausanne, Lausanne, Switzerland; 8 Department of Internal Medicine, Internal Medicine, Centre Hospitalier Universitaire Vaudois, University of Lausanne, Lausanne, Switzerland; 9 Division of Pediatric Endocrinology, Diabetes and Obesity, Centre Hospitalier Universitaire Vaudois, University of Lausanne, Lausanne, Switzerland; Weill Cornell Medical College in Qatar, QATAR

## Abstract

**Introduction:**

Low educational level (EL) and low physical fitness are both predictors of increased morbidity and mortality in patients with type 2 diabetes. It is unknown if EL is related to physical fitness. This would have important implication for the treatment approach of patients of low EL.

**Materials and Methods:**

In 2011/12, we invited participants of a new nationwide Swiss physical activity program for patients with type 2 diabetes to participate in this study. EL was defined by self-report and categorized as low (mandatory education), middle (professional education) or high (high school/university). Physical fitness was determined using 5 validated measures that assessed aerobic fitness, functional lower limb muscle strength, walking speed, balance and flexibility. Potential confounder variables such as other socio-cultural factors, physical activity level, body composition, diabetes-related parameters and complications/co-morbidities as well as well-being were assessed.

**Results:**

All invited 185 participants (mean age 59.6 ±9.8 yrs, 76 women) agreed to be included. Of all patients, 23.1% had a low, 32.7% a middle and 44.2% a high EL; 41.8% were professionally active. The study population had a mean BMI of 32.4±5.2 kg/m^2^ and an HbA1c of 7.3±1.3%. The mean diabetes duration was 8.8±7.4 years. In the baseline assessment, higher EL was associated with increased aerobic fitness, increased functional lower limb muscle strength, and increased walking speed using linear regression analysis (values for low, middle and high EL, respectively: 91.8 ± 27.9, 116.4 ± 49.7 and 134.9 ± 60.4 watts for aerobic fitness (p = 0.002), 15 ± 4.7, 13.9 ± 2.7, 12.6 ± 2.9 seconds for strength (p = 0.001) and 8.8 ± 1.6, 8.3 ± 1.4, 7.8 ± 1.4 seconds for walking speed (p = 0.004)). These associations were independent of potential confounders. Overall, aerobic fitness was 46%, functional limb muscle strength 16%, and walking speed 11% higher in patients of high compared to those of low EL. EL was not related to balance or flexibility.

**Discussion:**

A main strength of the present study is that it addresses a population of importance and a factor (EL) whose understanding can influence future interventions. A second strength is its relatively large sample size of a high-risk population. Third, unlike studies that have shown an association between self-reported fitness and educational level we assessed physical fitness measures by a quantitative and validated test battery using assessors blinded to other data. Another novelty is the extensive evaluation of the role of many relevant confounder variables.

**Conclusions:**

In conclusion, we show that in patients with type 2 diabetes EL correlates favorably and independently with important health-related physical fitness measures such as aerobic fitness, walking speed, and lower limb strength. Our findings underline that diabetic patients with low EL should be specifically encouraged to participate in physical activity intervention programs to further reduce social disparities in healthcare. Such programs should be structured and integrate the norms, needs and capacities (financial, time, physical capacities and self-efficacy) of this population, and their effectiveness should be tested in future studies.

**Trial Registration:**

University of Lausanne clinicaltrials.gov NCT01289587

## Introduction

Low socioeconomic status or educational level (EL) are risk factors for chronic diseases such as diabetes type 2, and related to worse metabolic control and diabetic complications in patients with diabetes [1]. Low EL is associated with a two-fold higher diabetes-related mortality as well as to a higher all-cause mortality [1,2]. In Switzerland, the prevalence of low EL (only mandatory school) is 15% among women and 10% among men [3].

Physical activity [4] and physical fitness measures such as aerobic fitness [5], global muscle strength [6] and walking speed [7] are negatively associated with mortality [8]. Physical activity improves physical fitness measures such as aerobic fitness, muscle strength, walking speed and balance in patients with type 2 diabetes [9]. In these patients, higher fitness is associated with improvements in cardiovascular risk factors such as glycemia, blood pressure [10], lipid profile and body composition [11], and with less functional decline [12]. Improving fitness thus represents a plausible goal for reducing morbidity and mortality in the diabetic population [8].

There is evidence for an impact of socioeconomic status on physical activity [13] and physical fitness measures [14]. For example, people with lower income are more likely to be physically inactive or less fit [15]. Academic achievements have been related to physical fitness in a young healthy population [14], and EL to self-reported physical fitness in a middle-aged healthy population [16]. However, it is unknown if this relationship holds true for patients with type 2 diabetes. If such a relationship is present, it would have clinical consequences regarding the encouragement and focus of educational modalities. Specifically, patient education programs, such as the nation-wide DIAfit program (www.diafit.ch), a program in Switzerland to promote regular exercise in patients with type 2 diabetes, should increase recruitment among low EL patients and adapt the program’s content to cover their needs with the aim to reduce morbidity and mortality.

Thus, the goal of this study was to investigate if EL is related to five physical fitness measures in patients with type 2 diabetes: aerobic fitness, functional lower limb muscle strength, walking speed, balance and flexibility. Given that such a relationship might be affected by important potential confounders, such as physical activity or body composition, these were assessed and adjusted for.

## Materials and Methods

### Subjects

The clinical DIAfit program was introduced in 2011 in the French-speaking part of Switzerland. All 185 diabetic patients that were referred to this program between 2011 and 2012 from all 11 existing DIAfit treatment centers in the French-speaking part of Switzerland were invited to participate in this study.

Inclusion criteria in the clinical DIAfit program patients were age ≥18 years and a diagnosis of type 2 diabetes for ≥3 months. Exclusion criteria were current diabetic foot ulceration; ischemia during an exercise test or state III peripheral vascular disease; untreated proliferative retinopathy and autonomic neuropathy with unstable blood pressure during the exercise stress test.

This current analysis is based on the cross-sectional data of the study. All patients underwent the same structured medical visit by physicians collaborating in the DIAfit program and who had a one-day training. Referring physicians also filled out a questionnaire regarding detailed information about the different diabetes complications (presence and type of retinopathy, microalbuminia or decreased renal function based on laboratory values within the last 3 months), presence, date of onset and extent of ischemic cardiac disease and peripheral arterial vascular disease), medical comorbidities and treatment. Patients had a fasting blood analysis, underwent physical fitness tests, wore a pedometer during three full days (Monday to Wednesday) and filled out questionnaires.

### Ethics

The study was approved by the ethical committee of Lausanne in 2011 (protocol 252/10) and all participants signed a written informed consent. The trial was registered (clinicaltrials.gov NCT01289587).

### Measures

#### Educational level and socio-cultural confounder variables

Educational level has been shown to be the strongest predictor of socioeconomic status [17] and is recognized as its proxy [18]. In addition, EL is attained early in life, is associated with income and occupation, can be measured easily and is rarely affected by subsequent health impairments [19]. Educational level was determined as the highest level of education. Low EL was defined as no education beyond mandatory school (9 years duration); middle EL as having had a professional education after mandatory school, and high EL as having had a high school or university degree [20]. Professional occupation was defined as active versus not active (retired, disabled or unemployed). Migrant status was defined by the country of birth (i.e., other than Switzerland).

#### Physical fitness

Physical fitness was assessed by five parameters: aerobic fitness, lower limb muscle strength, walking speed, balance and flexibility.

Aerobic fitness was evaluated with a maximal graded cycle ergometry test performed by a cardiologist blinded to the other data. Participants started at 20 Watts. Increments of 20 Watts per 2 min were made until exhaustion or until reaching one of the American College of Sports Medicine established criteria for maximal oxygen uptake [21]. Heart rate was continuously measured by ECG. Blood pressure and the rate of perceived exertion [22] were assessed at the end of each step. Recovery was monitored until heart rate was <100 bpm. The maximum achieved resistance (Watts) was retained for all calculations.

Lower limb muscle strength was assessed with the Chair Stand Test. Participants sat with arms folded across the chest and with their back against the chair. The patient was instructed to stand up and sit down five times as quickly as possible and the required time (sec) was recorded [23]. Walking speed was evaluated using the 10 m walking test; the participant walked at her/his preferred speed and the time (sec) was recorded [24]. Balance was evaluated with a single-leg balance test. Participants were instructed to maintain their balance on their preferred leg as long as possible. The test was stopped at 30 seconds and the maximal balance time (sec) was recorded [25]. Flexibility was measured using the finger-to-floor distance. After bending forward, the distance (cm) between the tip of the middle finger and the floor was measured [26]. All physical fitness tests were performed by one single evaluator (O.G.) who was blinded to the other data.

#### Confounding variables

Physical activity, foot deformity and falls: Physical activity, defined as number of steps per day, was recorded with the Yamax SW-200 digi Walker [27] during three full days (Monday to Wednesday). The mean number of steps per day over the three days was used for further analysis. Sports club participation was asked by questionnaire. Patients were asked if they are registered at one or more sport clubs to which they go on a regular basis.

The presence of feet deformations such as hallux valgus, claw or hammer toe, and presence of flat foot were assessed by visual inspection.

The number of falls within the last 12 months was reported during the medical interview or based on medical charts.

Body composition: BMI was calculated based on measured height and weight. Waist circumference was measured midway between the iliac crest and the lowest border of the rib cage. Body composition was assessed by bioelectrical impedance using a 4-polar single frequency device (RJL Systems, Model 101A; Detroit, MI, USA).

Diabetes-related parameters, presence or extent of diabetes complications: Information about diabetes duration, presence of retinopathy, nephropathy, ischemic cardiac disease, peripheral arterial vascular disease, and the current medical treatment were systematically retrieved from the referral letters, the existing medical charts, if the patient was referred from the same institution, or, if missing, collected during the medical DIAfit visit by asking the patient or the referring physician. Data were then reported in the medical charts. If data were missing or unclear regarding retinopathy, the patients’ ophthalmologists were also contacted and the presence of background, proliferative retinopathy and macular edema were noted. If the last exam was beyond 1 year, a repeat ophthalmology exam was requested. Regarding nephropathy, the presence of albuminuria or reduced glomerular filtration rate without any other etiology were used to define nephropathy. Albuminuria or plasma creatinine were tested during the visit, if no laboratory results performed within the last 3 months were provided by the referring physician or present in the chart. The presence of ischemic cardiac disease or peripheral vascular disease was diagnosed based on the report of the referring physician or the medical charts without performing another exam besides the standard cycle ergometry test done before and at the end of the program (see above). Vibration threshold was measured in every patient during the DIAfit medical visit using the mean value of the left and right foot measured by a graduated Rydel-Seiffer tuning fork (scale 0–8) applied to the respective hallux of each foot [28]. Of all complications, only the vibration threshold was directly measured by the DIAfit physicians in all patients and thus the other complications were only used for descriptive analyses.

Cardiovascular disease risk factors and well-being:Hypertension was defined as either treatment for hypertension or measured blood pressure values >140/90 mmHg [29]. Dyslipidemia was defined as TG >1.7 mmol/l, LDL >2.6 mmol/l or lipid-lowering treatment.

Well-being was assessed using the WHO Well-Being Scale (WBT-5) as used previously in a large diabetic population [30]. It is a generic global rating scale that measures subjective well-being, as the WHO considers positive well-being to be another term for mental health [31]. This score consists of 6 items on a Likert scale from 0 (never) to 5 (always). The total score is multiplied by 4, yielding a final score from 0 (worst thinkable well-being) to 100 (best thinkable well-being). Using a cut-off score of ≤50 (a score of ≤50 corresponds to a state of low well-being) as a screening for depression yielded a sensitivity and specificity of 86% and 81% respectively [31].

### Data analysis and Statistics

Statistical analyses were conducted using Stata 12 (Stata corp, College Station, TX, USA). Descriptive statistics were calculated as mean±SD for continuous variables, or percentage for categorical variables. Missing data were not imputed.

The associations between EL, coded as an ordinal variable from 1 (lowest education) to 3 (highest education), and the five physical fitness measures were assessed by least squares linear regression adjusting for age and sex.

The relationships between the following potential confounders and the five physical fitness measures were also assessed separately after adjusting for age and sex [32,33,34,35,36,37,38,39]: socio-cultural factors (professional occupation or migrant status), physical activity level, sports club participation, history of falls, presence of feet deformity, body composition (BMI, waist circumference, fat mass, lean mass), diabetes-related parameters and complications (diabetes duration and HbA1c (%), neuropathy as the only directly measured complication as well as cardiovascular disease factors (presence of hypertension) and well-being. If the significance level of any of these relationships was p<0.05, the association of EL with the specific fitness measures was adjusted for the respective confounders. Nonlinearity of the association was tested including quadratic terms of EL. Similarly, multicollinearity of the independent variables was tested using the Collin function of STATA. All VIF’s ranged between 1.8 and 4, suggesting lack of multicollinearity, as no VIF was over 10.

In order to identify the best modeling of fitness in this population, we also performed a backward-stepwise analysis on the five fitness measures including all above mentioned parameters as well as EL as predictors, keeping all terms with a significance level of p<0.1 and always including age and sex.

## Results and Discussion

### Description of population

**Fig 1:**
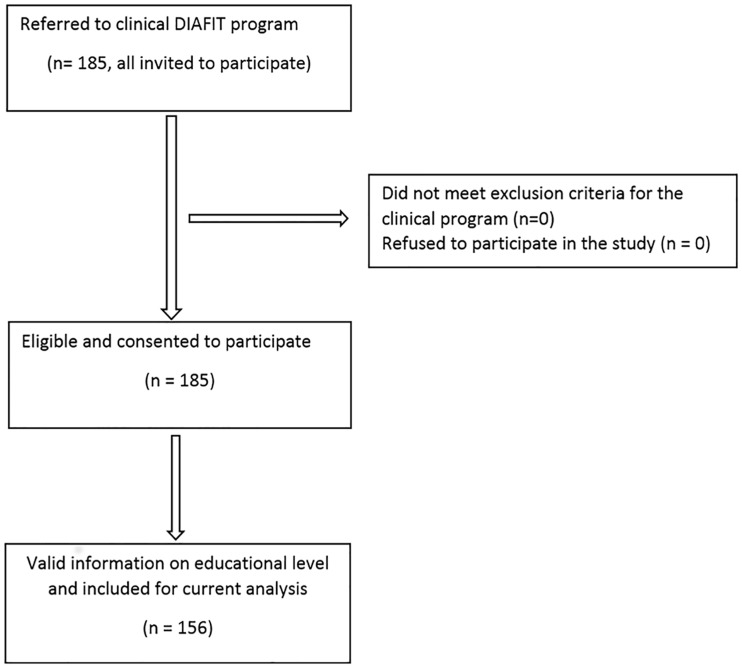
Description of the number of persons included and the number of patients used in our analysis.

All 185 recruited participants of the clinical DIAfit program agreed to participate in the study. One hundred fifty-six patients (76 women / 80 men, mean age 59.6±9.8 years) had a valid dataset with information on EL and represent the population that was used for the present analyses (Fig 1). Baseline characteristics are presented in Table 1. The demographic data as well as all fitness measures, except the walking speed (faster in those with available data on EL, p = 0.002), were similar in these 156 patients compared to the other 29 patients with missing data.

**Table 1:** Presentation of the baseline characteristics of the study population.

**General information**		
Age (years)	156	59.6 (9.8)
Gender (N of Females/ N of Males)	156	76/80
Educational level	156	
*Low*		36 (23.1%)
*Middle*		51 (32.7%)
*High*		69 (44.2%)
**Confounding variables**		
***Socio-cultural variables***		
Professional occupation	153	
Active professionally		64 (41.9%)
*0–20%*		*9 (14*.*1%)*
*25–50%*		*9 (14*.*1%)*
*55–75%*		*5 (7*.*8%)*
*80–100%*		*41 (64%)*
Non Active professionally		89 (58.1%)
* Retired*		*60 (39*.*2%)*
* Disabled* ^a^		*21 (13*.*7%)*
* Unemployed*		*8 (5*.*2%)*
Migrant status ^b^	152	63 (41.5%)
***Physical activity***		
* Number of steps/day*	102	6854.0 (3206.8)
* Sports club participation*	149	26 (17.5%)
***Presence of feet deformity***	122	33 (27.0%)
***Number of falls in last 12 months***	85	
*None*		74 (87.1%)
*One*		3 (3.5%)
*Two*		4 (4.7%)
*Three or more*		4 (4.7%)
***Body composition***		
Body weight (kg)	155	91.4 (17.8)
Body Mass Index (kg/m^2^)	155	32.4 (5.2)
* Normal weight*		*11(7*.*1%)*
* Overweight*		*44(28*.*4%)*
* Obesity*		*100(64*.*5%)*
Waist circumference (cm)	137	109.8 (11.7)
Fat body mass (kg)	140	35.3 (10.7)
Lean body mass (kg)	140	56.1 (13.9)
***Diabetes Related Parameters***		
Diabetes duration (years)	151	8.8 (7.4)
HbA_1c_ (%)	146	7.3 (1.3)
***Presence of diabetes complications***		
Presence of retinopathy	145	12 (8.3%)
Presence of nephropathy	138	14 (10.1%)
Neuropathy: Vibration Threshold (score 0–8)	152	5.6 (2.1%)
Presence of Ischemic cardiac disease	147	21 (14.3%)
Presence of peripheral arterial vascular disease	159	3 (1.9%)
***Presence of cardio-vascular disease risk factors factors***		
Presence of hypertension	154	122 (79.2%)
Presence of dyslipidemia	142	114 (74.5%)
***Other Parameters***		
Well-being (score)	127	57.6 (20.1)
* Low well-being* ^c^		40(31.5%)

^a^ Disability was defined by self-report for patients working 0–20% and report to receive assistance from the disability insurance (which can be for various medical reasons, but represent a social and medical vulnerability factor.

^b^ Defined as born outside of Switzerland

^c^ Low well-being is defined as a WHO wellbeing score of ≤ 50

Of all patients, 23.1% had a low, 32.7% a middle and 44.2% a high EL; 41.8% were professionally active. Forty-two percent of the study population were born outside of Switzerland. Patients had a mean BMI of 32.4±5.2 kg/m^2^ and a HbA1c of 7.3±1.3%. Their mean diabetes duration was 8.8±7.4 years. Microvascular (retinopathy, nephropathy or neuropathy) or macrovascular (ischemic heart disease or peripheral arterial vascular disease) diabetic complications were present in 2–14% of patients; 61% were treated with insulin, 24% with glucagon-like peptide-1 receptor agonists and 88% with oral anti-diabetic agents (56% of all patients were treated with metformin).

### Educational level and physical fitness

Table 2 illustrates the relationships of EL with the five physical fitness measures. Overall, participants achieved a mean power of 119.2±53.9 Watts in the aerobic fitness test, needed 13.6±3.4 sec to realize the lower limb strength test and 8.2±1.5 sec to walk a 10 m distance. They could stand on one leg during a mean of 19.8±10.7 sec and had a finger-floor distance (flexibility) of 11.4±9.7 cm.

**Table 2:** Relationships of educational level with physical fitness.

Fitness measures	N	Overall Mean(SD)	Low EL Mean(SD)	Middle EL Mean(SD)	High EL Mean(SD)	β-coefficient [95% CI]	p-value
Aerobic fitness (watts)	141	119.2 (53.9)	91.8 (27.9)	116.4 (49.7)	134.9 (60.4)	15.69 [5.96 to 25.42]	0.002
Lower limb strength (sec)	151	13.6 (3.4)	15.0 (4.7)	13.9 (2.7)	12.6 (2.9)	-1.17 [-1.88 to -0.47]	0.001
Walking speed (10m, sec)	152	8.2 (1.5)	8.8 (1.6)	8.3 (1.4)	7.8 (1.4)	-0.43 [-0.71 to -0.14]	0.004
Balance (sec)	152	19.8 (10.7)	19.5 (11.2)	19.0 (10.4)	20.5 (10.8)	0.13 [-1.85 to 2.11]	0.900
Flexibility (cm)	152	11.4 (9.7)	10.2 (7.7)	12.3 (10.0)	11.4 (10.5)	-0.55 [-2.51 to 1.40]	0.600

EL denotes Educational level. The associations between EL, coded as an ordinal variable from 1 (lowest education) to 3 (highest education), and the five physical fitness measures were assessed by least squares linear regression adjusting for age and sex.

There were no significant non-linear trends. A higher EL was related to (i) increased performance in aerobic fitness, (ii) increased functional lower limb muscle strength, and (iii) increased walking speed (all three p≤0.004). For these three fitness tests, this observed increase was gradual across the three EL categories. Thus, an increase from each EL to the next was associated with a mean adjusted increase of 15.7 Watts in aerobic fitness, a mean decrease of 1.2 sec in the time needed to perform the Chair Stand Test, and a mean decrease of 0.4 sec to walk a 10 meter distance. Overall, aerobic fitness was 46%, functional limb muscle strength 16%, and walking speed 11% higher in patients with high compared to those with low EL. EL was not related to balance or flexibility.

#### Potential confounding variables and physical fitness

In bivariate analysis, higher lean body mass and a higher vibration threshold were related to increased aerobic fitness (p ≤ 0.05). None of the confounders were related to lower limb strength. Being professionally or physically active was related to a higher walking speed (p ≤ 0.02). Longer diabetes duration and neuropathy (lower vibration threshold) were related to decreased balance performance (p ≤ 0.02). BMI, waist circumference and lean body mass were inversely associated with flexibility (p ≤0.03). All other tested associations between potential confounders and physical fitness measures were not significant.

Adjusting for the above mentioned significant confounder variables did not significantly alter the relationship between EL and aerobic fitness (n = 125, β-coefficient 15.7 (95%CI 5.3 to 26.0); p = 0.003 after adjusting for lean body mass and neuropathy) or EL and walking speed (n = 100, β-coefficient -0.5 (95%CI -0.8 to -0.1); p = 0.005 after adjusting for professional and physical activity).

#### Modeling of physical fitness

The results of the backward-stepwise analysis on the five physical fitness measures including all parameters are shown in Table 3.

**Table 3:** Modeling of physical fitness.

	Educational Level	Physical Activity level	Professional occupation	Vibration Threshold	Lean Body Mass	Fat Body Mass	Diabetes duration
		(N of 100 steps/day)	(non active vs active)	(0–8)	(kg)	(kg)	(years)
Aerobic fitness							
(watts; N = 141)	15.69						
adj R2 = 0.31	[5.96 to 25.42]						
p < 0.0001	p = 0.002						
Lower limb strength							
(sec; N = 139)	-1.35					0.06	
adj R2 = 0.09	[-2.08 to -0.62]					[0.05 to 0.12]	
p = 0.002	p<0.001					p = 0.04	
Walking speed							
(10msec;N = 149)	-0.401		0.882				
adj R^2^ = 0.20	[-0.69 to 0.12]		[0.39 to 1.37]				
p < 0.0001	p = 0.006		p < 0.001				
Balance							
(sec; N = 148)				1.50			
adj R2 = 0.30				[0.81 to 2.20]			
p < 0.0001				p<0.001			
Flexibility							
(cm; N = 140)					0.29		
adj R2 = 0.17					[-0.13 to 0.44]		
p < 0.0001					p<0.001		

Backward- stepwise analysis with the five fitness measures as outcome variable and socio-cultural variables, physical activity markers, feet deformity, number of falls within the last 12 months, body composition, diabetes-related parameters, extent of neuropathy, presence of cardiovascular disease factors and well-being, all of them after adjustment for age and sex as predictor variables. Adj R^2^ denotes adjusted R^2^ of the total model including age and sex. Data are shown as ß-coefficients [95% CI]. The associations between EL, coded as an ordinal variable from 1 (lowest education) to 3 (highest education), and the five physical fitness measures were assessed by least squares linear regression adjusting for age and sex.

While always including age and sex, aerobic fitness was best associated with EL. Walking speed was also associated with EL as well as with professional occupation (adjusted R^2^ 0.31 and 0.20, respectively, both p<0.0001). Lower limb strength was associated with EL and lower fat body mass. Better balance was associated with higher vibration threshold. Flexibility was associated with decreased lean body mass (respective adjusted R^2^ 0.09, 0.30, 0.17, all p≤0.002).

## Discussion

This observational study is based on evidence of a relationship between EL and mainly self-reported physical fitness in individuals without diabetes. It tests 1) whether this same relationship exists for individuals with type 2 diabetes, 2) whether it holds true for different measured fitness parameters and 3) whether a potential relationship is independent of confounder variables.

We have shown for the first time that in patients with type 2 diabetes higher EL is positively related to increases in aerobic fitness, functional lower limb muscle strength, and walking speed, three fitness measures that predict lower all-cause mortality in both the general and the diabetic population. In our population, a gradual increase in fitness was observed among the three EL categories. These relationships were independent of potential confounders such as other socio-cultural factors, physical activity, feet deformity, number of falls, body composition, diabetes duration and metabolic control, presence or extent of diabetes complications, presence of cardiovascular disease factors, and well-being.

The finding of a strong and independent relationship between EL and different physical fitness measures in patients with type 2 diabetes is clinically relevant. For example, the difference between low and high EL in aerobic fitness was 46%. Importantly, both higher EL and higher physical fitness have been shown to be related to reduced morbidity and mortality. This relationship might be due to a higher consciousness of the importance of fitness and the relationship between physical activity and health in subjects with increased EL. Furthermore, family activity habits differ according to EL [40]. However, other factors such as self-esteem, self-efficacy and quality of life might also play a role. Although tracking of physical fitness over several decades is only low- to moderate [41], we cannot completely exclude an impact of fitness on EL and on educational achievements in youth that might contribute to the current findings. This is especially the case as fitness has been found to be related to higher cognitive functioning [42].

The observed relationship between EL and physical fitness measures are in line with two previous studies conducted in healthy non-diabetic populations: Coe et al. [14] found an association between academic achievement and both aerobic fitness and muscular strength in healthy young students. Pullkinen et al. [16] conducted a study in Finland, with 2722 men and 3108 women aged from 25 to 74 years, focusing on the relationship between the level of education and self-rated physical fitness. A higher level of education was associated with better self-rated physical fitness measures. In addition, reported leisure-time physical activity was the strongest single explanatory factor for the educational differences in self-rated physical fitness measures [43].

In our study, EL was not related to balance and flexibility. One explanation might be that balance in this specific population is predominantly influenced by the severity of neuropathy [43]. In addition, usual physical activity exercises and recommendation for the general diabetes population focus more on endurance and resistance (leading to increased aerobic fitness and strength) than on balance or flexibility. This could have influenced our results [44].

Physical activity, body composition, diabetes duration, diabetic complications and professional occupation were also related to one or several fitness measures: Physical activity was related to aerobic fitness and walking speed in bivariate analyses. Increased lean body mass and less microvascular complications were related to aerobic fitness in bivariate analyses. This seems logical as a higher muscle mass is generally also related to a higher physical fitness [45], and because microvascular complications, such as peripheral neuropathy, limit weight-bearing exercise due to the loss of protective sensation in the feet. The identified relationship between the severity of neuropathy and balance has been previously shown [37]. Interestingly, higher BMI, waist circumference and lean body mass were inversely associated with flexibility. Higher (abdominal) body mass likely impaired the ability to reach towards the floor, while higher muscle mass can reduce the stretching ability of the hamstrings and calf muscles. We found that increased body fat mass was associated with decreased functional lower limb strength, when adjusted for EL. This may indicate that increased weight hinders a patient from standing up from a chair, even with normal analytical muscle strength. Having a structured daily life and thus more regular activity habits might explain why professionally active patients walked faster than those without professional activity. Although several factors were related to physical fitness, the found relationship between EL and aerobic fitness, strength and walking speed remained highly significant after adjusting for these potential confounders.

Overall, the results show that EL is independently related to physical fitness in patients with diabetes. Other factors such as number of steps per day, body composition and diabetic complications were also related to fitness. Based on these findings, the most important conclusion would be that a specific effort has to be undertaken to encourage physical activity promotion in this population. Structured physical-activity programs should be tailored in order to specifically target patients with low EL. The programs should increase knowledge about the important relationship between physical activity and health, as well as provide patients with tools to help them integrate physical activity in their daily life. Regarding other aspects of diabetes education, a recent study showed that a culturally sensitive, structured, group-based diabetes education based on theory of empowerment, food habits and health belief models can enhance biomedical and behavioral outcomes including patients with low EL [46]. The presence of complications should be taken into account in physical activity programs. For example, in patients with diabetic neuropathy, specific exercises should be included in order to improve balance and subsequently decrease their risk of falls. Indeed, static and dynamic balance as well as gait can be improved in this specific population [12]. In order to address body composition, physical activity is not sufficient, but should be complemented by nutritional approaches.

A main strength of the present study is that it addresses a population of importance in the health and a factor (EL) whose understanding can influence future interventions. A second strength is the fact that we recruited patients in non-specialized clinical centers. Importantly, all patients of the clinical program agreed to participate in the study which increases its representativeness considerably. The high prevalence of patients with diabetic complications and of patients that are treated with insulin further underlines its representativeness, as these patients are often excluded from trials related to physical activity or fitness. Only few data with a relatively large sample size of high-risk population do exist. Third, unlike studies that have shown an association between self-reported fitness and educational level we assessed physical fitness measures by a quantitative and validated test battery including five different fitness domains. A further strength is the extensive evaluation of many relevant known and potential confounder variables and predictors of fitness. One limitation is that we did not assess additional socio-cultural parameters such as income and wealth. Socioeconomic status is a rather complex construct usually held to comprise education, income, wealth and occupation [17]. In practice, wealth and income data are not always accessible to clinicians. Thus, we assessed EL, which is recognized as a stable proxy of socioeconomic status and easy to assess in the clinical setting. However, we only had valid information about the educational level for 156 of the 185 patients (84%). We further have to state that the relatively high percentage of subjects with a low EL might not be representative for the total Swiss population, but may be more representative for patients with type 2 diabetes. Also the cross-sectional design might be a limitation, as reversal causality could be a problem in interpreting data obtained. No a priori sample size calculation was performed for this outcome, because this is a sub-study of a randomized controlled trial assessing the impact of a physical activity intervention on aerobic fitness levels. Another consideration is that pedometers are not extremely precise measures of physical activity, and that physical activity was only measured over 3 days. In addition, information about past physical activity was not available. Therefore, we cannot completely exclude that physical activity did play an important role to explain the identified differences in fitness.

## Conclusion

In conclusion, we show that in patients with type 2 diabetes EL correlates favorably and independently with important health-related physical fitness measures such as aerobic fitness, walking speed, and lower limb strength. Our findings suggest that patients with diabetes and low EL should be specifically encouraged to participate in physical activity intervention programs to reduce social disparities in healthcare. Such programs should be structured and integrate the norms, needs and capacities (financial, time, physical capacities and self-efficacy) of this population, and their effectiveness should be tested in future studies.

## Supporting Information

S1 File Minimal data set underlying the findings of your manuscript. (DTA)

## Abbreviations

BMI: Body Mass Index
Cm: Centimeter
EL: Educational Level
Kg: Kilogram
N: Number
SD: Standard Deviation
Sec: Seconds
WHO: World Health Organization

## References

[1] Saydah S, Lochner K (2010) Socioeconomic status and risk of diabetes-related mortality in the U.S. Public Health Rep 125: 377–388. 20433032 10.1177/003335491012500306 PMC2848262

[2] Nilsson PM, Johansson SE, Sundquist J (1998) Low educational status is a risk factor for mortality among diabetic people. Diabet Med 15: 213–219. 10.1002/(SICI)1096-9136(199803)15:3<213::AID-DIA569>3.0.CO;2-# 9545122

[3] http://www.bfs.admin.ch/bfs/portal/fr/index/themen/20/05/blank/key/gleichstellung_und/bildungsstand.html.

[4] Paffenbarger RS Jr., Hyde RT, Wing AL, Lee IM, Jung DL, et al (1993) The association of changes in physical-activity level and other lifestyle characteristics with mortality among men. N Engl J Med 328: 538–545. 10.1056/NEJM199302253280804 8426621

[5] Snowden CP, Prentis J, Jacques B, Anderson H, Manas D, et al (2013) Cardiorespiratory fitness predicts mortality and hospital length of stay after major elective surgery in older people. Ann Surg 257: 999–1004. 10.1097/SLA.0b013e31828dbac2 23665968

[6] Lee JJ, Waak K, Grosse-Sundrup M, Xue F, Lee J, et al (2012) Global muscle strength but not grip strength predicts mortality and length of stay in a general population in a surgical intensive care unit. Phys Ther 92: 1546–1555. 10.2522/ptj.20110403 22976446

[7] Elbaz A, Sabia S, Brunner E, Shipley M, Marmot M, et al (2013) Association of walking speed in late midlife with mortality: results from the Whitehall II cohort study. Age (Dordr) 35: 943–952. 10.1007/s11357-012-9387-9 22361996 PMC3636402

[8] Wei M, Gibbons LW, Kampert JB, Nichaman MZ, Blair SN (2000) Low cardiorespiratory fitness and physical inactivity as predictors of mortality in men with type 2 diabetes. Ann Intern Med 132: 605–611. 10.7326/0003-4819-132-8-200004180-00002 10766678

[9] Allet L, Armand S, de Bie RA, Golay A, Monnin D, et al (2010) The gait and balance of patients with diabetes can be improved: a randomised controlled trial. Diabetologia 53: 458–466. 10.1007/s00125-009-1592-4 19921145 PMC2815802

[10] Umpierre D, Ribeiro PA, Schaan BD, Ribeiro JP (2013) Volume of supervised exercise training impacts glycaemic control in patients with type 2 diabetes: a systematic review with meta-regression analysis. Diabetologia 56: 242–251. 10.1007/s00125-012-2774-z 23160642

[11] Bacchi E, Negri C, Zanolin ME, Milanese C, Faccioli N, et al (2012) Metabolic effects of aerobic training and resistance training in type 2 diabetic subjects: a randomized controlled trial (the RAED2 study). Diabetes Care 35: 676–682. 10.2337/dc11-1655 22344613 PMC3308269

[12] Allet L, Armand S, Aminian K, Pataky Z, Golay A, et al (2010) An exercise intervention to improve diabetic patients' gait in a real-life environment. Gait Posture 32: 185–190. 10.1016/j.gaitpost.2010.04.013 20471273

[13] Mabry RM, Reeves MM, Eakin EG, Owen N (2010) Evidence of physical activity participation among men and women in the countries of the Gulf cooperation council: a review. Obes Rev 11: 457–464. 10.1111/j.1467-789X.2009.00655.x 19793376

[14] Coe DP, Pivarnik JM, Womack CJ, Reeves MJ, Malina RM (2012) Health-related fitness and academic achievement in middle school students. J Sports Med Phys Fitness 52: 654–660. 23187329

[15] Freitas D, Maia J, Beunen G, Claessens A, Thomis M, et al (2007) Socio-economic status, growth, physical activity and fitness: the Madeira Growth Study. Ann Hum Biol 34: 107–122. 10.1080/03014460601080983 17536760

[16] Pulkkinen K, Makinen T, Valkeinen H, Prattala R, Borodulin K (2013) Educational differences in self-rated physical fitness among Finns. BMC Public Health 13: 163 10.1186/1471-2458-13-163 23433081 PMC3639841

[17] Burgi F, Meyer U, Niederer I, Ebenegger V, Marques-Vidal P, et al (2010) Socio-cultural determinants of adiposity and physical activity in preschool children: a cross-sectional study. BMC Public Health 10: 733 10.1186/1471-2458-10-733 21110865 PMC3008696

[18] Speybroeck N, Konings P, Lynch J, Harper S, Berkvens D, et al (2010) Decomposing socioeconomic health inequalities. International journal of public health 55: 347–351. 10.1007/s00038-009-0105-z 20063112

[19] Lahana E, Pappa E, Niakas D (2010) The impact of ethnicity, place of residence and socioeconomic status on health-related quality of life: results from a Greek health survey. International journal of public health 55: 391–400. 10.1007/s00038-010-0171-2 20652355

[20] Burgi F, Niederer I, Schindler C, Bodenmann P, Marques-Vidal P, et al (2012) Effect of a lifestyle intervention on adiposity and fitness in socially disadvantaged subgroups of preschoolers: a cluster-randomized trial (Ballabeina). Prev Med 54: 335–340. 10.1016/j.ypmed.2012.02.007 22373886

[21] Medicine ACoS Guidelines for exercise testing and prescription. 7th Edition, Lippincott, Williams and Wilkins Editors.

[22] Borg GA (1982) Psychophysical bases of perceived exertion. Med Sci Sports Exerc 14: 377–381. 10.1249/00005768-198205000-00012 7154893

[23] Bean JF, Kiely DK, Herman S, Leveille SG, Mizer K, et al (2002) The relationship between leg power and physical performance in mobility-limited older people. J Am Geriatr Soc 50: 461–467. 11943041 10.1046/j.1532-5415.2002.50111.x

[24] Peters D, Fritz S, Krotish D (2013) Assessing the reliability and validity of a shorter walk test compared with the 10-Meter Walk Test for measurements. J Geriatr Phys Ther 36: 24–30. 10.1519/JPT.0b013e318248e20d 22415358

[25] Rossier P, Wade DT (2001) Validity and reliability comparison of 4 mobility measures in patients presenting with neurologic impairment. Arch Phys Med Rehabil 82: 9–13. 10.1053/apmr.2001.9396 11239279

[26] Katzmarzyk PT, Craig CL, Gauvin L (2007) Adiposity, physical fitness and incident diabetes: the physical activity longitudinal study. Diabetologia 50: 538–544. 10.1007/s00125-006-0554-3 17221212

[27] Crouter SE, Schneider PL, Bassett DR Jr. (2005) Spring-levered versus piezo-electric pedometer accuracy in overweight and obese adults. Med Sci Sports Exerc 37: 1673–1679. 10.1249/01.mss.0000181677.36658.a8 16260966

[28] Singh N, Armstrong DG, Lipsky BA (2005) Preventing foot ulcers in patients with diabetes. JAMA 293: 217–228. 10.1001/jama.293.2.217 15644549

[29] (2014) Standards of medical care in diabetes—2014. Diabetes Care 37 Suppl 1: S14–80. 10.2337/dc14-S014 24357209

[30] Heun R, Burkart M, Maier W, Bech P (1999) Internal and external validity of the WHO Well-Being Scale in the elderly general population. Acta Psychiatr Scand 99: 171–178. 10.1111/j.1600-0447.1999.tb00973.x 10100911

[31] Topp CW, Ostergaard SD, Sondergaard S, Bech P (2015) The WHO-5 Well-Being Index: a systematic review of the literature. Psychother Psychosom 84: 167–176. 10.1159/000376585 25831962

[32] Droomers M, Schrijvers CT, Mackenbach JP (2001) Educational level and decreases in leisure time physical activity: predictors from the longitudinal GLOBE study. J Epidemiol Community Health 55: 562–568. 10.1136/jech.55.8.562 11449013 PMC1731951

[33] Tulloch H, Sweet SN, Fortier M, Capstick G, Kenny GP, et al (2013) Exercise facilitators and barriers from adoption to maintenance in the diabetes aerobic and resistance exercise trial. Can J Diabetes 37: 367–374. 10.1016/j.jcjd.2013.09.002 24321716

[34] Miettinen ME, Kinnunen L, Leiviska J, Keinanen-Kiukaanniemi S, Korpi-Hyovalti E, et al (2014) Association of Serum 25-Hydroxyvitamin D with Lifestyle Factors and Metabolic and Cardiovascular Disease Markers: Population-Based Cross-Sectional Study (FIN-D2D). PLoS One 9: e100235 10.1371/journal.pone.0100235 25000408 PMC4085035

[35] Smith L, Hamer M (2014) Television viewing time and risk of incident diabetes mellitus: the English Longitudinal Study of Ageing. Diabet Med. 10.1111/dme.12544 24975987 PMC4236275

[36] Quddus MA, Uddin MJ (2013) Evaluation of foot ulcers in diabetic patients. Mymensingh Med J 22: 527–532. 23982544

[37] Allet L, Armand S, de Bie RA, Pataky Z, Aminian K, et al (2009) Gait alterations of diabetic patients while walking on different surfaces. Gait Posture 29: 488–493. 10.1016/j.gaitpost.2008.11.012 19138520

[38] Lee YJ, Hung WL (2011) The relationship between exercise participation and well-being of the retired elderly. Aging Ment Health 15: 873–881. 10.1080/13607863.2011.569486 21547748

[39] Stubbe JH, de Moor MH, Boomsma DI, de Geus EJ (2007) The association between exercise participation and well-being: a co-twin study. Prev Med 44: 148–152. 10.1016/j.ypmed.2006.09.002 17059845

[40] Tol A, Mohebbi B, Sadeghi R (2014) Evaluation of dietary habits and related factors among type 2 diabetic patients: An innovative study in Iran. J Educ Health Promot 3: 4 10.4103/2277-9531.127548 24741644 PMC3977396

[41] Soric M, Jembrek Gostovic M, Gostovic M, Hocevar M, Misigoj-Durakovic M (2014) Tracking of BMI, fatness and cardiorespiratory fitness from adolescence to middle adulthood: the Zagreb Growth and Development Longitudinal Study. Ann Hum Biol 41: 238–243. 10.3109/03014460.2013.851739 24200353

[42] Aberg MA, Pedersen NL, Toren K, Svartengren M, Backstrand B, et al (2009) Cardiovascular fitness is associated with cognition in young adulthood. Proc Natl Acad Sci U S A 106: 20906–20911. 10.1073/pnas.0905307106 19948959 PMC2785721

[43] Turcot K, Allet L, Golay A, Hoffmeyer P, Armand S (2009) Investigation of standing balance in diabetic patients with and without peripheral neuropathy using accelerometers. Clin Biomech (Bristol, Avon) 24: 716–721. 10.1016/j.clinbiomech.2009.07.003 19683372

[44] ADA (2014) Standards of Medical Care in Diabetes. Diabetes Care 37.

[45] Lee CD, Blair SN, Jackson AS (1999) Cardiorespiratory fitness, body composition, and all-cause and cardiovascular disease mortality in men. Am J Clin Nutr 69: 373–380. 10075319 10.1093/ajcn/69.3.373

[46] Mohamed H, Al-Lenjawi B, Amuna P, Zotor F, Elmahdi H (2013) Culturally sensitive patient-centred educational programme for self-management of type 2 diabetes: a randomized controlled trial. Prim Care Diabetes 7: 199–206. 10.1016/j.pcd.2013.05.002 23830727

